# Editorial: Spotlight on resistant and refractory migraine

**DOI:** 10.3389/fneur.2023.1291439

**Published:** 2023-09-27

**Authors:** Raffaele Ornello, Bianca Raffaelli

**Affiliations:** ^1^Department of Biotechnological and Applied Clinical Sciences, University of L'Aquila, L'Aquila, Italy; ^2^Department of Neurology, Headache Center, Charité Universitätsmedizin Berlin, Berlin, Germany

**Keywords:** resistant migraine, refractory migraine, migraine prevention, migraine treatment, clinical trials, real-world evidence, experimental model

Migraine is the second cause of disability worldwide and the first in young women (1). While migraine affects more than one billion people worldwide (2), it manifests with a wide spectrum of impact on individuals. This ranges from infrequent occurrences to chronic forms that severely disable those affected.

Migraine management traditionally relies on acute treatments to halt attacks when they occur and on preventive agents to avert future episodes. Failure of migraine preventive treatments is a frequent challenge in clinical practice (3). Many conventional preventive agents were initially designed for other medical conditions and subsequently applied to migraine, resulting in limited effectiveness and poor tolerability (4). The landscape shifted with the introduction of a new generation of migraine-specific treatments designed to antagonize calcitonin gene-related peptide (CGRP) and its receptor. These treatments have achieved high adherence rates and the possibility of long-term persistence due to their improved tolerability (5). In large clinical trials (6–9) and in real-world studies (10, 11), monoclonal antibodies against CGRP and the CGRP-receptor have proven effective even in patients who were previously considered resistant to traditional preventive treatments.

This transition to new migraine-specific preventive treatments prompted a redefinition of the concept of refractory migraine. In 2020, the European Headache Federation revised the definitions of resistant and refractory migraine. According to these updated definitions, migraine is considered “resistant” if an individual experiences at least 3 months of eight or more debilitating headache days monthly, despite attempting at least three classes of preventive medication, which have proven ineffective, intolerable, or contraindicated. When an individual experiences at least 6 months of eight or more debilitating headache days per month and has failed all drug classes of preventive treatment, including CGRP-targeted drugs, the condition is labeled “refractory” (12). These definitions distinguish between conditions that, although disabling, can still benefit from advanced migraine prevention (resistant migraine) and those in which drug treatment is exceedingly challenging (refractory migraine). While less common in the general population, patients with resistant migraine are encountered frequently, at least weekly, in three-quarters of headache centers, while those with refractory migraine are encountered at least weekly in half of these centers (13).

Resistant and refractory migraine are an important object of migraine research due to their representation of the most severe forms of the disorder and their role in understanding migraine pathophysiology and perpetuating factors. The introduction of new definitions may further boost efforts to comprehend the biology of resistance to treatments. This Research Topic provides a comprehensive overview of the diverse range of study designs dedicated to understanding resistant and refractory migraine, encompassing both clinical and preclinical research.

Beginning with a preclinical perspective, Zhang et al. examined fibrinogen levels and vestibular dysfunction in a mouse model of chronic migraine induced by nitroglycerin exposure. The authors observed increased fibrinogen levels and reduced performance in vestibular tests following nitroglycerin application, suggesting that chronic head pain can induce alterations in the central nervous system and even impact coagulation. While chronic migraine itself does not necessarily equate to resistance to preventive treatments, continuous exposure to head pain in chronic migraine can lead to central sensitization to pain (14), potentially resulting in increased resistance to preventive treatments.

A particularly resistant form of chronic migraine is linked to the overuse of acute medication. Research has demonstrated that continuous exposure to high doses of analgesics not only diminishes the efficacy of these drugs but can also exacerbate headaches (15). The introduction of novel migraine-specific medications can be beneficial in reversing the condition of medication overuse (16). To demonstrate the effectiveness of anti-CGRP treatments in chronic migraine with medication overuse, a randomized placebo-controlled clinical trial is currently underway—the RESOLUTION trial involving the use of eptinezumab in patients with chronic migraine and medication overuse headache (Jensen et al.). Eptinezumab, an anti-CGRP monoclonal antibody, is administered intravenously every 12 weeks. The RESOLUTION trial comprises both a placebo-controlled phase and an open-label phase of eptinezumab administration, alongside a counseling intervention known as the Brief Intervention. The aim is to reduce acute medication consumption and manage medication overuse through combined pharmacological and non-pharmacological measures. RESOLUTION started in July 2022 and is supposed to be completed in May 2024.

Monoclonal antibodies targeting the CGRP pathway represent a significant breakthrough in migraine treatment; however, they are not a universal solution. Patients who exhibit resistance to monoclonal antibodies offer an intriguing avenue for investigating the pathophysiology of resistant and refractory migraine, given that monoclonal antibodies are specifically designed to target a pathogenic mechanism of migraine. Additionally, it is worthwhile to explore whether differences exist among monoclonal antibodies, potentially attributable to variations in pharmacokinetics, pharmacodynamics, chemical structure, and interindividual differences in drug metabolism. Two pieces of evidence included in this Research Topic address the switch between different monoclonal antibodies. The report by Overeem et al. explored the switch from antibodies binding to the CGRP molecule (fremanezumab and galcanezumab) to the antibody binding to the CGRP receptor (erenumab). Erenumab was the first licensed monoclonal antibody acting on the CGRP pathway, and previous reports primarily focused on switching between anti-receptor and anti-ligand antibodies. The study by Overeem et al. examined the opposite journey, i.e., from anti-ligand to an anti-receptor antibody. Remarkably, the effectiveness results were comparable to those obtained in studies involving switches between erenumab and anti-ligand antibodies (17–20). Around 30% of patients showed a positive response, defined as ≥30% reduction in monthly headache days, after switching to another antibody class. Switching between antibody classes might therefore be a valuable solution in clinical practice if patients do not respond to the first CGRP(-receptor) antibody. The second paper (Uzun et al.) presented a case study of a patient with chronic migraine treated with all three monoclonal antibodies—erenumab, fremanezumab, and galcanezumab. This patient had initially reported constipation with erenumab and was compelled to switch from fremanezumab to galcanezumab for several months due to logistical reasons. Intriguingly, the patient exhibited distinct responses to all three antibodies and displayed varying tolerability profiles. This case study suggests that monoclonal antibodies targeting the CGRP pathway, despite their shared focus on the same pathway, may exhibit different efficacy and tolerability profiles.

An essential factor to consider in understanding resistant and refractory migraine is the role of comorbidities. Migraine is a complex biopsychosocial disorder wherein individual predisposition interacts with comorbidities and psychosocial factors that can either improve or worsen the condition (21). While the role of comorbidities is well-acknowledged in the transformation of migraine into a chronic disorder (22), their exact influence on resistance to migraine treatments remains an ongoing area of exploration.

Depression is a widely recognized comorbidity of migraine, closely intertwined with migraine-related disability (23). Within our Research Topic, a study explored the issue of comorbidity between depression and migraine, specifically focusing on genetic aspects through a Mendelian randomization approach (Lv et al.). Leveraging data from a genome-wide association study, the authors identified major depression as a risk factor for migraine, with the reverse association being less likely. This study offered an interesting perspective on the relationship between depression and migraine, highlighting shared genetics, particularly among the most severe forms of depression and the most severe forms of migraine.

In conclusion, our Research Topic provided a comprehensive overview of recent innovations across various study designs within the realm of resistant and refractory migraine research. This area continues to evolve, particularly in the era of accessible migraine-specific preventive treatments. The collaboration between preclinical and clinical research in this field promises to yield insights into migraine pathophysiology and the discovery of new treatment targets.

## Author contributions

RO: Conceptualization, Writing—original draft, Writing—review and editing. BR: Conceptualization, Writing—original draft, Writing—review and editing.
